# Methylation and demethylation of DNA and histones in chromatin: the most complicated epigenetic marker

**DOI:** 10.1038/emm.2017.38

**Published:** 2017-04-28

**Authors:** Hong-Duk Youn

**Affiliations:** 1 Department of Biomedical Sciences, National Creative Research Center for Epigenome Reprogramming Network, Seoul National University College of Medicine, Seoul, Korea; 2 Department of Molecular Medicine & Biopharmaceutical Sciences, Graduate School of Convergence Science, Seoul National University, Seoul, Korea

Epigenetics is defined as heritable changes in genomic function that occur without changes in the DNA sequence. Accumulating evidence has revealed that epigenetics is critical in understanding the basic molecular mechanisms that take place in chromatin (transcription, splicing, replication and DNA repair), and epigenetics is ultimately expected to provide insights into complicated biological processes such as stem cell maintenance and development and be applied to human diseases, including cancers.

Chromatin is bound by a diverse group of proteins that dynamically regulate its structure by modifying, reading and erasing its basic components—histones and DNA. In particular, DNA and histones are primarily post translationally modified through various enzymatic activities, such as phosphorylation, acetylation, methylation, ubiquitination and O-GlcNAcylation. Combinations of these epigenetic modifications regulate chromatin dynamics by preparing specific loci for activation or repression ultimately governing the global chromatin architecture with regard to gene expression during the cell cycle and development.

Methylation is the most complicated epigenetic modification. Unlike other changes, methylation alone affects both DNA and histones: methylation occurs primarily at the C^5^-cytosine and N^6^-adenosine in DNA and at lysine and arginine residues in histones and chromatin-binding proteins. Further, there are three forms of methylation on lysine residues—monomethyl, dimethyl, and trimethyl. For arginine, monomethylated, symmetric and asymmetric dimethylated forms appear. Each form has a specific function, rendering it difficult to determine the purpose of their combinations. Also, the position of the affected lysine (or arginine) in the amino-acid sequence of histones influences its epigenetic function because methylation at various positions is programmed to recruit cognate reading proteins for repression or activation. Thus, methylation of DNA and histones is the most advanced epigenetic marker in the regulation of chromatin dynamics.

In this special issue of *Experimental & Molecular Medicine*, we focus on the regulation of cytosine methylation on DNA and lysine methylation of histones in stem cell pluripotency and cancer development.

Mirang Kim (KRIBB, Korea) and Joseph Costello (University of California, San Francisco, USA) review DNA cytosine methylation as an epigenetic marker of cellular memory. 5-Methylcytosine (5 mC) was initially discovered as a minor base that was introduced into the progeny strand during DNA replication. Later, a family of DNA methyltransferases (DNMT1, DNMT3a/b/l) was discovered to catalyze the methylation of the 5-carbon of cytidine in CpG dinucleotides. They also introduce an advanced method for analyzing DNA cytosine methylation. By developing DNA methylation analysis and genomewide sequencing tools, we now understand that DNA cytosine methylation is dispersed throughout chromatin and serves as an epigenetic marker of memory in the development, differentiation and maintenance of cellular identity. Further, they discuss the epigenetic function of cytosine methylation in various types of stem cells and cancer cells.

In the second issue, Myunggon Ko’s group (UNIST, Korea) presents a comprehensive review on DNA demethylation in stem cells and cancer. DNA cytosine methylation has been considered a stable epigenetic modification, but the pioneering discovery by Anjana Rao’s group (La Jolla Institute for Allergy and Immunology, USA) that ten-eleven translocation 1 (TET1) oxidizes 5 mC to 5-hydroxymethylcytosine (5 hmC) confirms that cytosine methylation is dynamically modulated during stem cell differentiation and development. Notably, three isoforms of TET can catalyze the successive oxidation of 5 mC to 5 hmC, 5-formylcytosine (5 fC) and 5-carboxylcytosine (5 caC), which differs from the reversible demethylation of methylated lysines in histones. It will be interesting to study why mammalian cells adopt a more complicated reactive pathway for 5 mC demethylation. In addition, the authors discuss the involvement of TET in cancers. Loss of TET function is common in various cancers, and thus the in-depth study of TET-mediated DNA cytosine oxidation should increase our understanding of how epigenetic dysregulation causes tumorigenesis.

Histone lysine methylation is more complex than cytosine methylation in DNA. Thus, Jaehoon Kim’s group (KAIST, Korea) summarizes all known enzymes that are involved in lysine methylation and demethylation and their functions in various processes. Several lysine methyltransferases catalyze methylation marks on chromatin loci, to which cognate proteins are recruited and eventually determine epigenetic function. Conversely, demethylases remove these marks to reset loci for second-round methylation or simply antagonize the methylation signal. Many methyltransferase-demethylase pairs have been identified in mammals, but no H3K79 demethylase has been discovered. The finding of an H3K79 demethylase will be necessary to understand the complete pairing system between histone lysine methylation and demethylation.

Fei Lan’s group (Fudan University, China) focuses on the epigenetic function of histone demethylases in embryonic development. They first discuss how lysine demethylases regulate epigenetic states at specific genomic loci, including bivalent promoters, enhancers and repetitive elements, because the methylation state of these chromatin elements is linked to cell fate decisions. They also review the epigenetic function of histone lysine demethylases in various processes during embryonic development.

In the final article, TaeSoo Kim’s group (Ewha Womans University, Korea) and Stephen Buratowski (Harvard Medical School, USA) discuss the cotranscriptional mechanism by which histone methylation at H3K4 and H3K36 regulates transcription in cooperation with other histone epigenetic markers, such as acetylation and mono-ubiquitination. For instance, H3K4me3 and histone acetylation are enriched in the promoters of genes. H3K36me2/me3 are found preferentially in gene bodies, in which Eaf3-mediated recruitment of HDACs effects the deacetylation of histones during transcription. Given that cotranscriptional regulation of histone methylation and acetylation has been linked to many cancers, more detailed research in this field will help identify cancer-related genes and apply this to anticancer drug development.

In this special issue of *Experimental & Molecular Medicine*, five review articles summarize the general concepts of methylation of histones and DNA and provide an overview of the current information on how methylation regulates stem cell maintenance, differentiation and cancer development.

